# The Sound of Scotoma: Audio Space Representation Reorganization in Individuals With Macular Degeneration

**DOI:** 10.3389/fnint.2019.00044

**Published:** 2019-08-20

**Authors:** Hafsah Ahmad, Walter Setti, Claudio Campus, Elisabetta Capris, Valentina Facchini, Giulio Sandini, Monica Gori

**Affiliations:** ^1^Robotics, Brain and Cognitive Sciences, Italian Institute of Technology, Genoa, Italy; ^2^Unit for Visually Impaired People, Italian Institute of Technology, Genoa, Italy; ^3^Department of Informatics, Bioengineering, Robotics, and Systems Engineering, University of Genoa, Genoa, Italy; ^4^Istituto David Chiossone, Genoa, Italy

**Keywords:** macular degeneration, multi-sensory integration, scotoma, audio-space representation, PRL

## Abstract

Blindness is an ideal condition to study the role of visual input on the development of spatial representation, as studies have shown how audio space representation reorganizes in blindness. However, how spatial reorganization works is still unclear. A limitation of the study on blindness is that it is a “stable” system and it does not allow for studying the mechanisms that subtend the progress of this reorganization. To overcome this problem here we study, for the first time, audio spatial reorganization in 18 adults with macular degeneration (MD) for which the loss of vision due to scotoma is an ongoing progressive process. Our results show that the loss of vision produces immediate changes in the processing of spatial audio signals. In individuals with MD, the lateral sounds are “attracted” toward the central scotoma position resulting in a strong bias in the spatial auditory percept. This result suggests that the reorganization of audio space representation is a fast and plastic process occurring also later in life, after vision loss.

## Introduction

**FIGURE 1:**
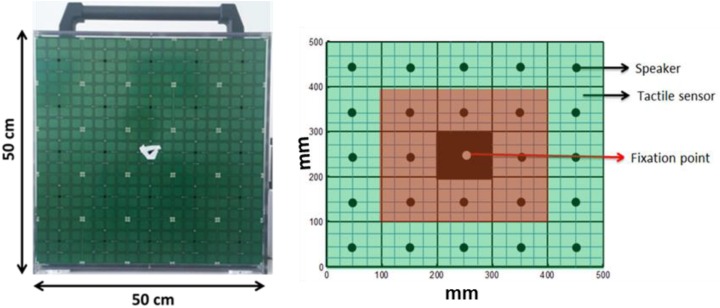
The device and simulation of device.

In sighted individuals, the visual cortex responds mainly to visual inputs. Recent evidence shows that in some specific cases the visual cortex of blind individuals processes spatial information of audio and tactile signals (Rauschecker, 1995; Collignon et al., 2009, 2011, 2013; Voss and Zatorre, 2012). Moreover, sighted individuals are reported to show a reset in visual cortex driven by auditory phase shifts and this kind of cross modal changes is found extensively in visual cortex (Mercier et al., 2013; Keil and Senkowski, 2018). This result is in agreement with studies in sighted individuals showing multisensory interactions between sensory modalities in human primary cortices (Martuzzi et al., 2006; Romei et al., 2009). This cortical reorganization in blindness has been associated with the enhanced abilities of blind individuals in processing audio information such as sound localization in the azimuth location (Lessard et al., 1998; Voss et al., 2004; Röder et al., 2007). However, blind individuals are not always better in the audio processing than sighted individuals and in some cases they show strong impairments  in  audio  space  representation  tasks

such as in the spatial bisection task or in the dynamic sound localization (Gori et al., 2014; Finocchietti et al., 2015; Vercillo et al., 2015). To date, it is not clear why some skills are enhanced and some other impaired. More in general, an open question is the start of cortical and perceptual reorganization after the beginning of the visual impairment. A limit of the study of blindness is that it is a “stable” system and it does not allow for study of the mechanisms that subtend the progress of cross-sensory plastic changes. To overcome this problem we studied, for the first time, audio spatial reorganization in individuals with macular degeneration (MD) for which the loss of vision due to scotoma is an ongoing progressive process. MD is a retinal disorder that damages the retina and produces scotoma (blind spots) on the eye cutting inputs on corresponding visual cortical representations (Sunness et al., 1996; Hassan et al., 2002; Schuchard, 2005). MD is an ideal condition to study the mechanisms that subtend audio spatial reorganization. Depending upon the pathology, scotoma can be central or peripheral, hereditary (also called “juvenile” JMD), or age-related (AMD). More in general, retinal damage increases with time and thus the scotoma size. 18 MD individuals with central visual scotoma were involved in an audio spatial task. Auditory stimuli were presented at different points of the frontal surface consisting of a vertical matrix of speakers, considering spaces within (central), and outside (peripheral) the visual scotoma (see Figure 1 for details). Our hypothesis was that if the lack of vision has a direct and immediate effect on the cross-modal reorganization of spatial audio representation, this should provide a distortion of audio processing within the scotoma zone in MD but not in sighted individuals. Our results support our hypothesis showing that the loss of vision produces changes in the processing of spatial audio signals in MD patients. In individuals with MD, the lateral sounds are “attracted” toward the central scotoma position resulting in a strong bias in the spatial auditory percept. We discuss our results suggesting that the reorganization of audio space representation is a fast and plastic process occurring in a few years also later in life, starting after vision loss.

## Materials and Methods

### Subjects

A total of 18 MD participants (mean age: 66.28, standard deviation: 21.74) and 18 sighted subjects (mean age: 53.72, standard deviation: 19.55), unpaired *t*-test (*t* = 1.58, df = 33.55, *p* = 0.12), and participated in the study (see details in Table 1). We performed a power analysis based on data acquired in pilot studies and we estimated for the difference between groups, an effect size (measured with Cohen’s d) which were at least 0.96 (large according to Cohen’s classification). Based on the expect size, on a significance of 0.05 and a statistical power of 0.8, we retained as sufficient a minimum sample of approximately 18 subjects All MD participants were suffering from central vision loss due to scotoma caused by different diseases as reported in Table 1. Some of these participants were born with congenital retinal diseases (JMD, e.g., RP) leading to slow degeneration of the retina and development of central scotoma with growing age, while others were suffering from AMD; hence developing a scotoma in one or both eyes in later years of life. All these patients were recruited from “Istituto David Chiossone” based in Genoa, Italy. Since all these participants were suffering from central vision loss (central scotoma), they were part of a rehabilitation program where they were learning to fixate with their preferred retinal locus (PRL) instead of damaged fovea using certain rehabilitation training techniques. All necessary subject data (history, visual acuity, disease, dominant eye, PRL, fixation, and retinal maps) were obtained from the ophthalmologist and rehabilitators at “Istituto David Chiossone” as shown in Table 1 (visual acuities for P06, P16, P17, and P18 are not reported in the table, as the hospital was unable to provide a VA record for these participants). The dominant eye of sighted participants was determined prior to the experiment using the classic dominant eye test (Heiting, 2017).

**TABLE 1:** Characteristics of MD participants.

			**Duration of**				**Dominant**
**ID**	**Age (Y^∗∗^)**	**Disease**	**disease (Y^∗∗^)**	**Visual acuity**			**eye**
				**Left^∗^**	**Right^∗^**	**Both**	
P01	83	Glaucoma	15	1/20	1/20	1/20	Right
P02	87	AMD	03–04	1–2/10	Blind	1–2/10	Left
P03	86	Myopia + Maculopathy	02	Blind	1/15	1/15	Right
P04	85	Myopia	15	1/10	Blind	1/10	Left
P05	18	Maculopathy + RP	Congenital	1	1–2/10	1	Right
P06	62	AMD	15	–	–	–	Right
P07	77	Maculopathy + AMD	15	1/100	1/10	1/10	Right
P08	75	Maculopathy + AMD	10	1/20	1/10	1/10	Right
P09	82	Maculopathy + AMD	20	1/50	1/100	1/50	Left
P10	80	AMD	30	1/20	Blind	1/20	Left
P11	22	RP	Congenital	1/20	Blind	1/20	Right
P12	70	AMD	05	1/10	1/100	1/10	Left
P13	78	AMD	07–08	1/20	1/20	1/20	Right
P14	78	Myopia	20	1/20	Blind	1/20	Left
P15	73	AMD	10	1/50	Blind	1/50	Left
P16	42	Maculopathy	03	–	–	–	Left
P17	51	Glaucoma	26	–	–	–	Right
P18	44	JMD	08	–	–	–	Right

*^∗^Left/Right Eye; ^∗∗^Years.*

### Ethics Statement

All subjects involved in this study were adults (age above 16 years). This study was approved by the ethics committees of the local health services: Comitato Etico, ASL3 Genovese, Italy. Subjects (both patients and controls) signed the written informed consents prior to performing the experiment.

### Stimuli and Procedure

A 5 × 5 matrix (dimension 50 cm × 50 cm) of 25 speakers (each speaker dimension 10 cm × 10 cm) was used for the experiment. Each speaker was covered by 16 haptic blocks, making the whole matrix touch-sensitive (see Figure 1). Sounds were produced using sound card of PC and controlled using Matlab R2013b^®^ (MathWorks.Inc.).

Before starting the experiment, fixation stability and a retinal map of each patient were obtained using the Nidek MP-1 Retinal Microperimetry (NIDEK TECHNOLOGIES SRI) with the help of a rehabilitator at “Istituto David Chiossone.” The retinal images provided by microperimetry covered a visual angle of ±20 degrees (essentially where the central scotoma was present). Since all the MD participants had vision loss due to central scotoma, device matrix was virtually divided into central and peripheral parts as shown in Figure 1. The red highlighted part mimics the center of the eye (covering a visual angle of ±23.7 degrees) while the green highlighted part mimics the periphery (covering visual angle of ±47.47 degrees). None of the subjects were aware of the virtual division of the matrix. Subjects sat straight at a distance of 30 cm from the device with their eyes positioned in front of the fixation point in the center of matrix (see Figure 1). Position of device was adjusted according to height of subject.

The experiment was divided into two conditions; Monocular and blindfolded. All subjects (MD participants and sighted) performed the test in the Monocular condition, while only a sub-group of participants (9 MD participants and 8 sighted subjects) performed the experiment in blindfolded condition as well. This subgroup was estimated using power analysis based on pilot studies for the difference between groups in the blindfolded condition, an effect size (measured with Cohen’s d) which was at least 1.5 (large according to Cohen’s classification). Based on the expect size, on a significance of 0.05 and a statistical power of 0.8, we retained as sufficient a minimum sample of approximately 8 subjects. The blindfolded condition was tested on a sub-group of participants that performed the major study in order to check if there is a bias due to visual inputs or not. In the monocular condition, subjects were asked to fixate (with dominant eye) at the marked fixation point in the center of the device while listening to sounds produced from different speakers (white noise, duration 1 s). Participants were asked to touch, with the index finger of the dominant hand, the position from where they perceived sound was produced, hence localizing the sounds, while fixating at the center of the device. Here it is important to mention that MD participants were asked to fixate with their PRL, while controls were asked to fixate with their fovea. When the touch was registered by the tactile sensors, a feedback sound (“meow” of a cat) was reproduced from the central speaker to end the trial. Thus, the subject was allowed to bring his/her finger back to resting position. A pause of 3 s was inserted between trials. A total of 72 random trials were produced with each speaker producing sound 3 times randomly (central speaker marked as fixation point only produced feedback sound). The same experiment was repeated in the blindfolded condition while blindfolding both eyes and localizing sounds. A training session was also run until subject understood the task before starting of actual experiment.

### Subject Responses

**FIGURE 2:**
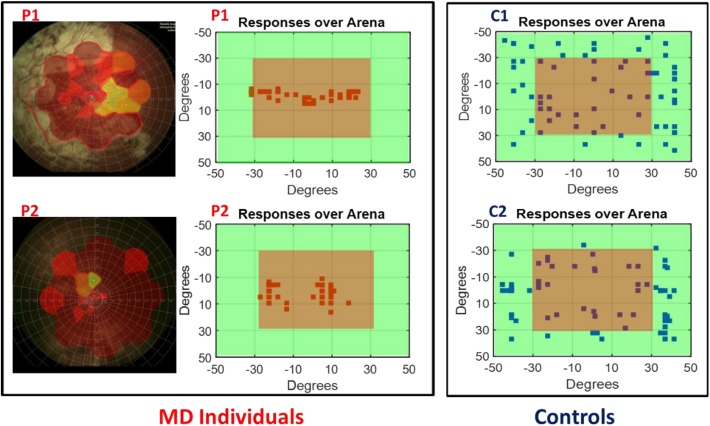
Subject responses. Left, example of retinal maps for two MD individuals (P1 and P2). The central red area indicates the damaged retina, yellow for partially damaged retina, and green for the leftover healthy part of the retina. Center, example of the responses of the same two MD individuals (P1 and P2) for sound localization. Sounds were equally distributed on the surface of the device, but their responses were mainly localized in the central region (in red) indicating the position of their scotoma. Right, example of the responses of two age sighted participants (C1 and C2) for sound localization. Responses are equally distributed on the surface.

To determine the scotoma position, the fixation stability of subjects and the exact visual angle subtended by the scotoma, we collected retinal maps (Chen et al., 2009) for all the MD participants (see Figure 2 left for an example of retinal maps in two participants). Subject responses were recorded over the device matrix and are shown as a function of visual angles in relation with the fixation point on the device. As an example, in Figure 2 (central panel) are provided responses of the two MD participants (whose retinal maps are presented on the left) and for two sighted individuals. While for sighted individuals (Figure 2, blue dots) the responses for sound localization are equally distributed on the surface, the responses of the MD participants (Figure 2, red dots), were mainly localized on the central region, namely where the scotoma was present suggesting an “attraction” of sound toward the scotoma position.

## Results

To quantify the sensory precision and the bias in sound localization (i.e., the sound attraction toward the scotoma position), responses were subdivided as central responses (CR) and peripheral responses (PR), considering the central and peripheral portions of the device (Figure 1B), respectively.

**FIGURE 3:**
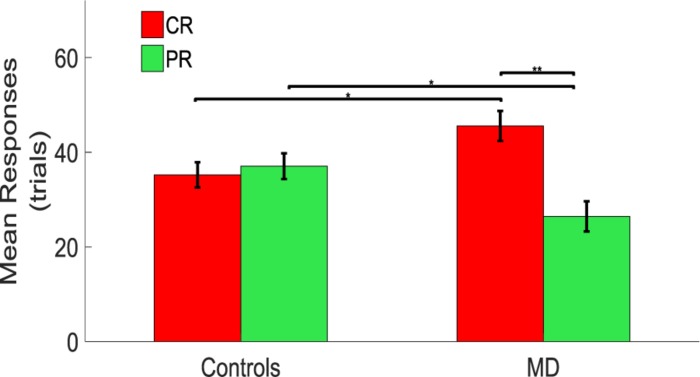
Comparison between controls and MD considering CR and PR. Results show that the MD participants (right side) are more attracted toward the central speakers (red bars). This attraction is higher compared to the one showed by the sighted (left side) which provided responses equally distributed for central and peripheral regions. ^∗^Significance between groups; ^∗∗^Significance with-in group.

A significant difference between CR and PR was found in MD participants with a higher number of responses in the CR than in the PR. A mixed model ANOVA (2 × 2) was performed with the group as between factor (two levels, sighted and MD), and position as within factor (two levels, CR and PR). A significant interaction was found between group and position [*F*(1,34) = 6.79, *p* = 0.02]. *Post hoc t*-tests revealed that MD individuals tend to touch the central speakers (CS) more compared to the sighted individuals (MD: mean = 45.56, SEM = 3.18, Controls: mean = 34.72, SEM = 2.67, un-paired *t*-test, *t* = 2.58, df = 33.01, *p* = 0.014), while sighted participants tend to touch the peripheral speakers more compared to the MD individuals (MD: mean = 26.45, SEM = 3.18, Controls: mean = 37.56, SEM = 2.72, un-paired *t*-test; *t* = −2.65, df = 33.19, *p* = 0.012). Also, MD individuals touched more the central rather than the peripheral speakers (CR: mean = 45.56, SEM = 3.18; PR: mean = 26.45, SEM = 3.18, paired *t*-test: *t* = 3.01, df = 17, *p* = 0.008). Sighted participants respond equally in the CR and PR (CR: mean = 34.72, SEM = 2.67; PR: mean = 37.56, SEM = 2.72, paired *t*-test: *t* = −0.53, df = 17, *p* = 0.61) as shown as a bar plot in Figure 3.

**FIGURE 4:**
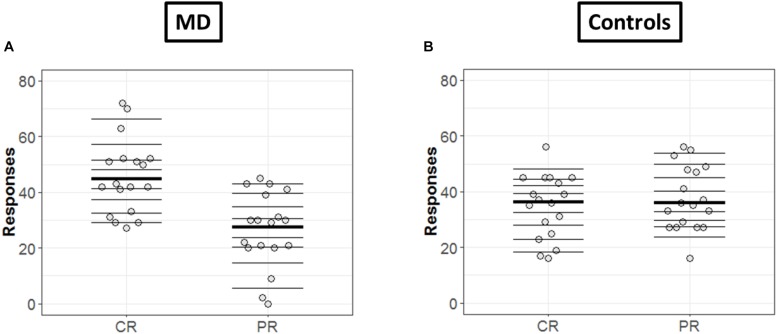
Differences in CR and PR for MD **(A)** and controls **(B)** groups. **(A**,**B)** Strip chart of two distributions. Each circle represents one participant, horizontal lines shows the deciles and thicker line show the median. The dotted line corresponds to zero.

In order to get a detailed picture of how CR are comparable to PR, we implemented in R the methods developed by Rousselet et al. (2017). First, we extracted all the deciles and medians of distributions in each condition (CR and PR) and for each group (MD and controls) as shown in Figures 4A,B, respectively. The horizontal lines represent the nine deciles with a thicker line showing the median of each condition, the dots represent each participant.

**FIGURE 5:**
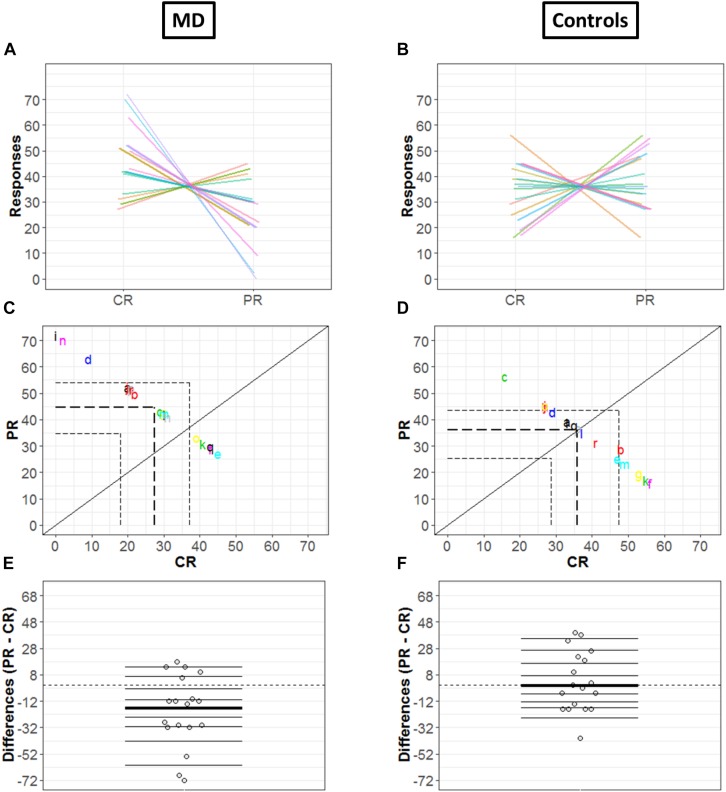
Differences in CR and PR for MD (left panel) and controls (right panel) groups. **(A**,**B)** Pairwise observations. Paired observations of each subject are joined by a single line of a different color. **(C**,**D)** Scatter plot. The diagonal black line shows reference with no effect; CR = PR (slope = 1, intercept = 0). Colored letters show the scattered data points and dashed line show quartiles for each condition. **(E**,**F)** Strip chart of difference responses. Each circle represents the difference between conditions for one participant. Deciles are shown by horizontal lines; the thicker line shows the median.

Since the two conditions (CR and PR) are paired, the investigation was not merely limited to computation of marginal distributions; we also computed how responses are linked between center and periphery for MD (Figure 5A) and Controls (Figure 5B) group, respectively: paired observations of each subject are joined by a single line of a different color. Figure 5A show that a majority of lines are decreasing from CR to PR, suggesting a greater tendency for responding in the center compared to the periphery, while Figure 5B reveals the absence of any trend due to a huge variability among the slopes of subjects.

Figures 5C,D also show the link between two conditions in terms of decile differences, the thicker line represents the difference in medians for two conditions. The black diagonal shows line of no effect with slope one and intercept zero as reference line (CR = PR). Quartiles of two conditions are shown by the dashed lines. Here, it is important to mention that since the total number of trials is constant (i.e., 72), CR and PR are negatively related (CR = 72 – PR). This means that if a subject responds more in the center (CR), the value of PR automatically reduces and vice versa, hence a negative correlation between CR and PR. For the MD group, Figure 5C shows differences that are quite scattered from the center. Whereas for controls, Figure 5D shows that the differences are rather symmetrically grouped around the central line revealing that the probability of having subjects with positive or negative differences between conditions are similar.

Figures 5E,F illustrate the distribution of the differences between CR and PR. The horizontal lines show the deciles with the thicker black line showing the median of differences. Difference between marginal distributions of CR and PR is larger for MD than for control groups. In fact, for MD group, the median for CR is 42.5 and for PR it is 29.5. The difference between the two medians is −13 with a 95% confidence interval of (−68.6, 14.6) (Figure 5E). Figure 5F shows the differences between marginal distribution (CR: median = 36.5; PR: median = 35.5) for the control group as strip charts. The difference between the two medians is +1 with a 95% confidence interval of (−21.3, 38.3).

**FIGURE 6:**
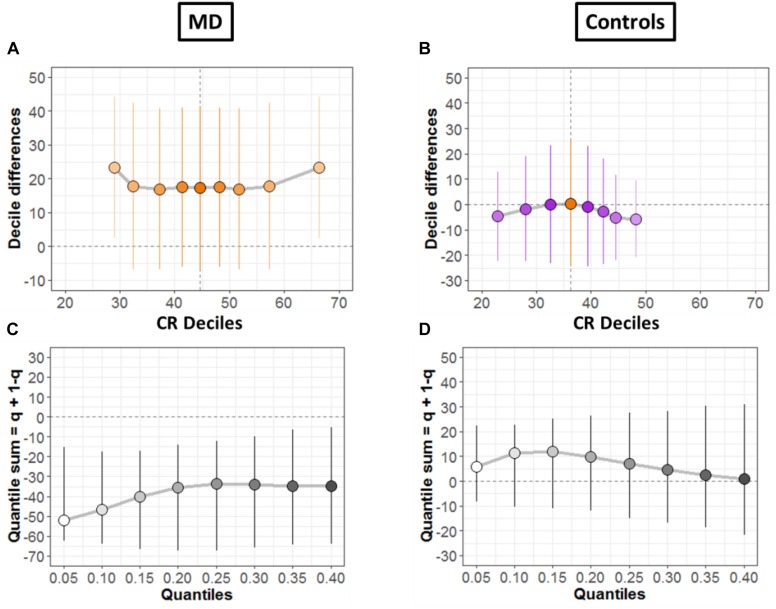
Differences between conditions. **(A,B)** Shift function with 95% confidence intervals. **(C,D)** Difference asymmetry function with 95% confidence intervals computed via bootstraps technique.

To systematically compare the distributions, shift function for dependent variables was also evaluated (Doksum, 1974; Wilcox and Erceg-Hurn, 2012), as shown for both groups in Figures 6A,B, respectively. The circles represent the decile differences and the vertical lines correspond to the 95% confidence interval which is computed using bootstrap technique (2000 bootstrap samples) (Rousselet et al., 2017). The vertical dashed line shows the mean. For each decile, confidence intervals which are not crossing zero correspond to significant difference. For the MD group (Figure 6A) only the first and the last decile differ significantly. Instead, for the controls group in Figure 6B, we see no significant difference for any decile.

Then, we quantified distribution difference asymmetries using a new method called difference asymmetry function, proposed by Wilcox (Wilcox and Erceg-Hurn, 2012). The method computes the quantile sums = q + (1 – q) considering different quantile estimations by using Harrell-Davis estimator. The confidence intervals are derived using the percentile bootstrap technique. Figures 6C,D show the resulting difference asymmetry function for MD and Controls groups, respectively. Along *x*-axis, the starting point 0.05 shows the sum of quantile 0.05 + quantile 0.95; the next point 0.10 is for the sum of quantile 0.10 + quantile 0.90; and continues along the axis in similar fashion. MD group (Figure 6C) show negative sums at extreme quantiles (0.05 + 0.95) for all deciles. On the other hand, the controls group (Figure 6D) show that distributions do not differ because the confidence intervals difference asymmetry function is crossing zero line for all deciles.

**FIGURE 7:**
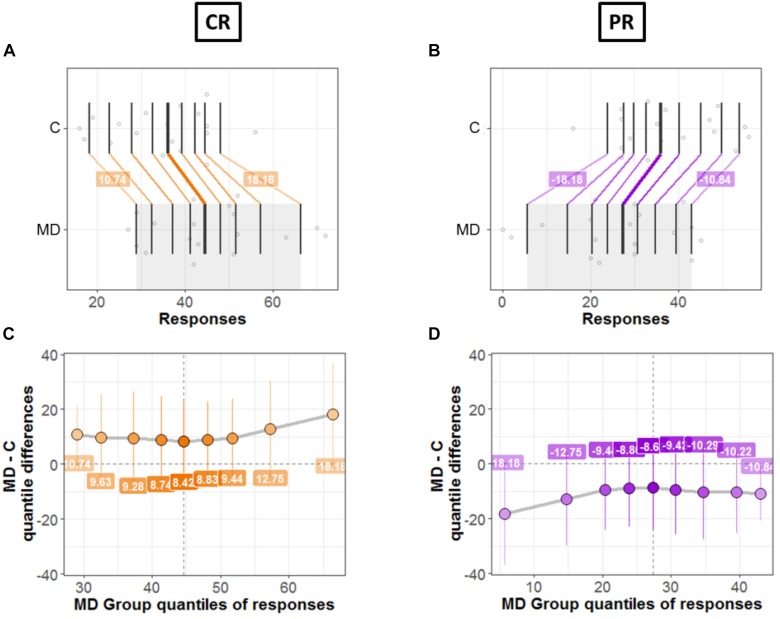
Differences between groups; MD and Controls for CR (left panel) and PR (right panel). **(A**,**B)** Strip charts for marginal distributions. Vertical lines mark the deciles for each group with a thicker line marking the median. Among distributions, the colored lines join the matching deciles (orange for positive decile differences and purple for negative values). **(C**,**D)** Shift function. Decile differences are shown with MD group deciles on *x*-axis and decile difference (MD-C) on *y*-axis. The vertical lines show the 95% bootstrap confidence interval. The first and the last deciles in both figures do not cross zero, hence they are considered significant.

**FIGURE 8:**
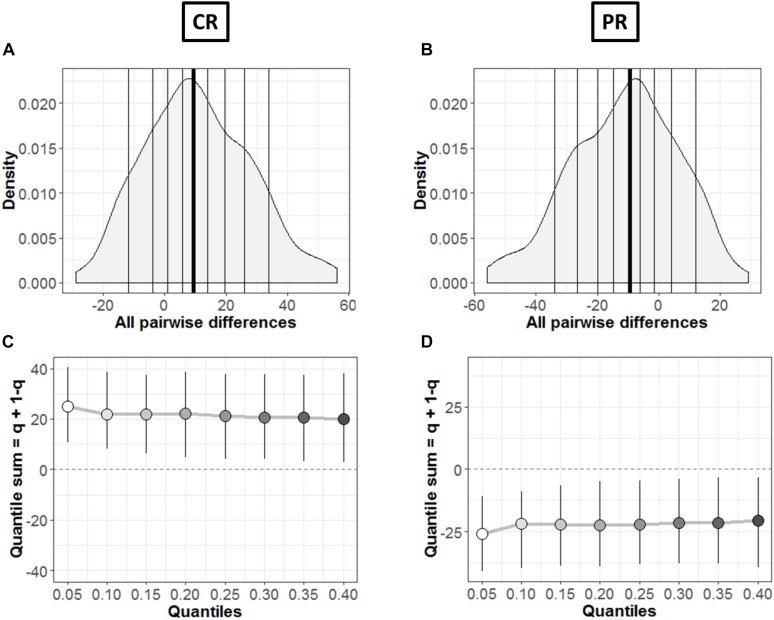
Comparison between groups. **(A**,**B)** Kernel density depiction of the distribution of all pairwise differences amongst the two groups. Deciles are marked by vertical lines with a thicker line for median. **(C**,**D)** Difference asymmetry function using 95% confidence intervals. The pair-wise error is controlled by altering the critical *p*-values with Hochberg’s method; the confidence intervals are not adjusted.

Next, we compared the bias for each condition (CR and PR) between the two groups (MD and controls). Figures 7, 8 shows a detailed picture of comparison between MD and C (controls) group for CR (left Panel) and PR (right Panel), respectively.

Figures 7A,B show the two marginal distributions in the form of a strip chart for each condition (CR and PR), respectively. The spread of the dots for each group (MD and C) is proportional to the local density of responses recorded for the said condition (CR or PR). The vertical lines show the deciles for each group with the thicker line showing the median of distributions. For instance, Figure 7A shows the distributions for two groups when the responses were recorded in the center (CR). For MD, the median of responses is 42.5 and for C median is equal to 36.5; hence the marginal difference is +6. As can be seen in Figure 7A, there is a shift between the distributions of the two groups: the deciles of MD are systematically greater compared to the C group. The difference in deciles is positive and is represented by orange lines joining corresponding deciles for each group. Decile values for first and ninth decile are +10.82 and +17.67, respectively as shown in Figure 7A. Similarly, Figure 7B shows the marginal distributions in the similar fashion as that of Figure 7A but for the PR condition. It is evident from Figure 7B that the shift between distributions is opposite in PR condition compared to CR condition, as expected because CR = 72 – PR. MD group is shifted to lower values (median = 29.5 and controls have higher values median = 35.5). The difference in the medians is −6 and the corresponding deciles are joined by purple lines showing a negative shift. This means that MD participants show dominance in CR condition compared to Controls and vice versa for PR condition.

Figures 7C,D shows the shift function for each condition, respectively. In both figures, on *x* axis we have deciles for MD which correspond to the gray shaded area in Figures 7A,B. Instead, on the *y*-axis we have the decile differences (MD – C). Hence, for each decile the shift function shows by how much one observation needs to be shifted to match another one. The vertical lines show the 95% bootstrap confidence interval. Only the first and the last deciles in both figures do not cross zero, hence they are considered significant.

In order to find the typical differences between the members of the two groups (MD and C), Figures 8A,B shows the kernel density representation (Han et al., 2004) of pairwise differences for each condition, respectively. The number of participants in each group is 18 (*n* = 18), so we get a total of 324 differences. In Figure 8A, the median of the differences is 9.49 i.e., far from zero with a 95% confidence interval at (2.39, 18.98). Hence, if we randomly select a sample from each group, it will differ significantly (Rousselet et al., 2017). These differences are distributed asymmetrically; negative values extend around −30 while positive values extend around −57. So, positive differences out-weigh negative differences in this case; revealing that the two differences differ. Similarly, Figure 8B shows this difference in case of PR condition. The median of differences is −9.57 with a 95% confidence interval at (−19.15, −3.23), which is – as expected – again far from zero. The asymmetry is also evident with negative values extending to −57 and positive values extending to +30, again showing an opposite behavior to CR condition with pairwise differences.

The difference asymmetry method introduced earlier for dependent conditions (Figures 6C,D) is also applied in this case for the two groups in each condition, respectively. Figures 8C,D shows the resulting difference asymmetry function for CR and PR, respectively. Along *x*-axis, the starting point 0.05 shows the sum of quantile 0.05 + quantile 0.95; the next point 0.10 is for the sum of quantile 0.10 + quantile 0.90; and continues along the axis in similar fashion. Condition CR (Figure 8C) shows always positive quantile sums (0.05 + 0.95). On the other hand PR (Figure 8D) shows again the opposite pattern with quantile sums below zero.

To disambiguate whether the effect was just a bias in response to the unseen area we tested the blindfolded condition. A sub-portion of individuals were taken from the groups of sighted and MD individuals (*N* = 8 and *N* = 9, respectively) as a control condition. Since the hypothesis of normality was not confirmed in this case, an ANOVA test is performed based on permutations by means of the R function *aovp* (Wheeler and Torchiano, 2010). The model (2 × 2 × 2) is provided by a between factor, *group* (MD and sighted), and two within factors: *condition* (monocular and blindfolded) and *position* (CR and PR). Only one significant interaction *group ^∗^ position* [*F*(1,59), *p* = 0.008)] is found, therefore, we performed *Post hoc* analysis with both paired and un-paired *t*-tests based on permutations as well (perm *t* test R function) (Fellows, 2012). Bonferroni correction is used for multiple comparisons. The only significant difference is found between the *positions* for MD participants (*t* = 3.71, df = 33.25, *p* = 0.003). The results show a higher tendency for MD individuals in touching the CS (coinciding with the position of the scotoma) compared to the group of the sighted even in blindfolded condition.

**FIGURE 9:**
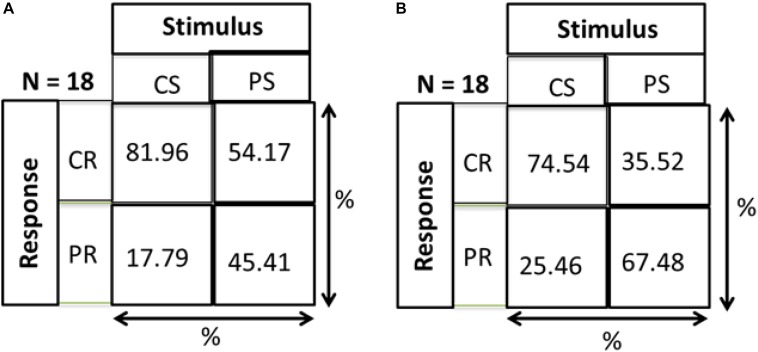
Hits and misses chart. CS, central stimuli; PS, peripheral stimuli; CR, central stimuli; PR, peripheral stimuli. The values are represented as percentage; For CS, total number of responses corresponding to CS/Total number of trials for CS; For PS, total number of responses corresponding to PS/Total number of trials for PS. **(A)** MD group. **(B)** Control group.

As a check that responses of both groups are a result of stimulus and not just random responses over the device, we calculated distance errors. Distance error is the distance between stimulus position and response position. We found that for central stimuli, the distance errors for MD and control groups are 9.86 and 9.74 cm, respectively, while in the periphery the distance errors are 15.8 and 14 cm, respectively. As mentioned in section “Materials and Methods” and shown in Figure 1, the distance between two speakers on the device is 10 cm. Hence, for both conditions the distance error is within 15 cm showing that responses correspond to stimuli and are not random. As evidence that subjects actually responded to the stimulus and didn’t make random responses on the device, a Hits and Misses matrix was computed for the two groups. Figure 9 shows the matrix computed to evaluate the percentage of responses. CS and PS represent the Central Stimulus and Peripheral Stimulus, respectively while CR and PR represent the CR and PR, respectively. The 2 × 2 matrix show the responses against the stimuli in terms of percentage. Percentage for CS (first column) is computed as the total number of responses when the sound was produced from CS divided by the total number of trials in the center (9 speakers × 3 trials each = 24). Similarly, the percentage value for PS (second column) is computed as the total number of responses when sound was produced in the periphery divided by total number of trials in the periphery (16 × 3 = 48). For instance, index (1,1) of the matrix shows the percentage of responses when both the stimulus and response were central, index (2,1) shows the percentage of responses when the stimulus was central but the response was peripheral, index (1,2) is the case when the stimulus was peripheral but the response was central and lastly, index (2,2) is the case when both stimulus and response were peripheral. Figure 9A represents that MD participants had a higher percentage to respond in center for central stimulus compared to Controls group (Figure 9B). The higher accuracy for the MD group can be explained in terms of results drawn from Figures 3–8. Since this group has a higher tendency to respond in the center, they have a higher probability to respond to central stimulus. This can also be explained in terms of peripheral stimuli. The percentage to respond correctly for peripheral stimuli is lower in MD compared to controls because MD group respond more frequently in the center. The same is true for incorrect responses as well. For the MD group, the percentage of correct responses in the center is almost double to the percentage of correct responses in periphery, which confirms the dominance to respond in the center. For controls group, the percentage of correct responses are almost equal, again as an evidence that they are not attracted toward any specific region, hence they are equally probable for correct and incorrect responses.

**FIGURE 10:**
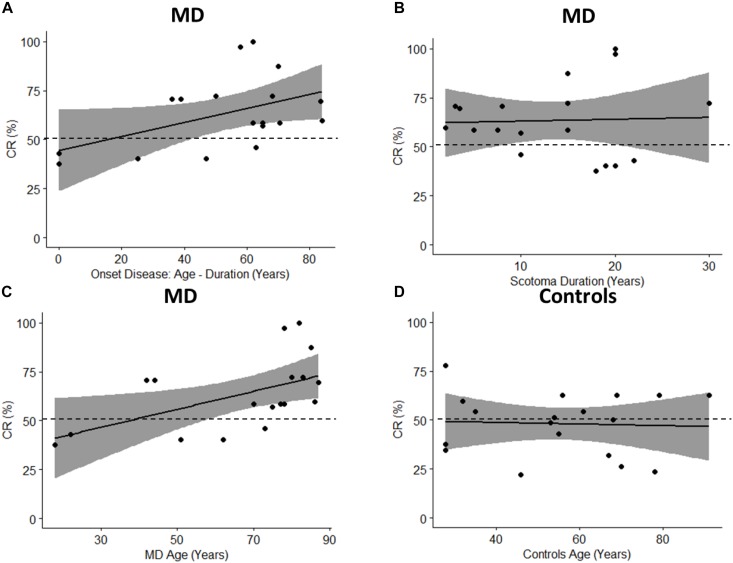
Pearson’s Correlations. The black dots represent data points; the black solid line represents regression line, the black dashed line shows 50% and the gray area show 95% confidence interval. **(A)** Correlation between onset age (age – duration) and percentage of CR (CR/72 ^∗^ 100). **(B)** Correlation between scotoma duration and percentage of CR. **(C)** Correlation between age of MD group and percentage of CR. **(D)** Correlation between age of controls group and percentage of CR.

To fully take advantage of MD as a model for audio-spatial representation and to provide more information about the mechanisms of multisensory recalibration we have analyzed the correlation between blindness duration and sound attraction. This correlation is analyzed by defining two parameters: Percentage of CR: which is calculated as CR/72 ^∗^ 100 (where 72 is the total number of trials); and the onset of scotoma that indicates when the scotoma was diagnosed in the first instance (Table 1); it is equal to the difference between the age and duration of the scotoma (for how long the subject has had the scotoma). A positive trend in correlation (Pearson’s coefficient *r* = 0.47, *p* = 0.051) is found between the Percentage of CR and the onset of the scotoma (Figure 10A). Results suggest that there is a trend in correlation between attraction toward the scotoma (CR) and clinical onset of the scotoma. Another correlation is computed between the Percentage of CR and duration of scotoma (*r* = 0.04, *p* = 0.88). As we have no significant correlation with the duration of disease, this shows that the effect remains consistent even when the duration increases (Figure 10B). The same result is confirmed by another correlation in which we considered the Percentage of CR against the age of MD individuals (Figure 10C) and the Percentage of CR against the age of typical participants (Figure 10D). A significant correlation between age and CR is evident only for MD individuals (Pearson’s coefficient (*r* = 0.53, *p* = 0.02) and not for typical (Pearson’s coefficient (*r* = 0.05, *p* = 0.94). The presence of an effect for the correlation of Age and CR for MD group and not for Controls group shows that MD participants are attracted more to the scotoma position with increasing age and that the correlation is present only when there is a “scotoma,” without scotoma (controls) we found no correlation.

## Discussion

Audio space reorganization was studied here for the first time in adults with central scotoma due to MD disease. Results suggest a robust attraction of sound toward the scotoma position in MD patients. Lateral sound positions were strongly biased and perceived as coming from the central scotoma region. The similar precisions in central and peripheral regions between MD and sighted participants (distance errors) suggest that the bias was not due to a less reliable spatial perception in MD individuals. Moreover, for MD participants the sound attraction toward the center is present even with eyes closed. On the contrary, there is no attraction toward a specific area of the device in controls both with eyes open or closed. This result indicates that the audio bias in MD individuals is not due to an attraction toward the unseen area supporting the idea of an ongoing multisensory recalibration process.

Results also support the idea that spatial reorganization of audio processing is an ongoing process that occurs after the loss of visual input in a plastic manner. The correlation that we observed between the attraction toward the center and onset of scotoma, indicates that the older the subject is at the onset of the scotoma, the more s/he is attracted toward the center. As expected, this result suggests that this multisensory recalibration process reflects the brain plasticity that is maximal in younger individuals and reduced at older ages (Lund, 1985; Kramer et al., 2004). This is even more interesting if we think that 12 of the 18 subjects tested were older than 70 years and the correlation effect between age and percentage of responses in the center was found only in the MD group and not in the control group. This suggests that central blind region has a minimal effect on audio–spatial reorganization of younger MD individuals, thanks to their cortical plasticity, and this effect due to scotoma increases in elderly population as cortical plasticity reduces with age (Erickson et al., 2007; Kramer and Erickson, 2007). Why do MD participants show an attraction of sound toward the central visual field, where they have the scotoma? Which is the mechanism associated with the bias we observed?

The ability to detect the spatial coordinates associated with neural signals from different sensory modalities is fundamental for a coherent perception. Given the superiority of visual over other sensory systems for space representation (Alais and Burr, 2004), the visual modality might offer a spatial background for remapping other sensory information. Supporting this idea, evidence suggests that eye-centered coordinates are used to align neural representations of space for different sensory modalities in the brain (Jay and Sparks, 1984; Cohen and Andersen, 2002; Pouget et al., 2002; King, 2009). When the visual information is not available, such as in blind individuals, the visual input starts to be activated by auditory stimuli and responses in these areas to auditory stimuli appear to be organized in a topographic manner (Rauschecker, 1995; Collignon et al., 2009, 2011, 2013; Voss and Zatorre, 2012; Abboud and Cohen, 2018; Harrar et al., 2018; Voss, 2018).

A possible explanation of our findings could be that the bias we observed is the result of the ongoing audio cortical reorganization due to the lack of visual input. This cortical reorganization is a fast process that starts immediately when the visual input is loss such as in MD individuals. The recruitment of the visual cortex from the auditory modality could produce the misperception of sound localization that we observed because audio and visual spatial maps require some time to realign. On the other hand, it is not clear which is the short term benefit of this audio reorganization. Indeed on one side, the attraction of sound is not useful to enhance audio spatial precision as it happens in blind individuals [as previously showed by Lessard et al. (1998)] since the audio precision we observed in this work is the same between sighted and MD participants. On the other side, it produces a strong misperception of sound, which is perceived as more central than the real position and this can be problematic for MD individuals.

Taking into consideration these two aspects mentioned above, a second possible explanation that we can consider is that the effect observed here is a result of multisensory integration process. Spatial audio and visual information are commonly integrated to create a unique percept when vision is available. In sighted individuals, given the higher reliability of the visual information for space, a visual dominance is reported as for example in the ventriloquist effect (as predicted by Bayesian Modeling e.g., see Alais and Burr, 2004). Considering this processing, our results could be also discussed in terms of reorganization of multisensory mechanisms. When the high reliability of visual input is decreasing, due to the loss of visual input such as in MD participants, the remaining visual spots are more weighted than predicted. This wrong weight may affect the spatial processing of multisensory information resulting in a capture of sound thus producing an “inverse ventriloquist effect.” This effect could be stronger in older than young participants who show less cortical plasticity and less multisensory integration skills (Lund, 1985; Kramer et al., 2004) which is in agreement with our correlation results.

Thirdly, a final possibility is that attention may have a role on the bias we observed. Santangelo and Macaluso (2012) have reviewed several behavioral and fMRI studies showing that attention can affect how audio and visual signals interact with each other in spatial domain (Santangelo and Macaluso, 2012; Stein, 2012). In this context, scotoma is indeed a “black hole” and with potential risks coming therefore, attentional resources can act as anchors by attracting audio signals in the invisible regions to increase the quantity of information, hence drawing attention of audio modality toward the non-visual zone. To disentangle which one of these three explanations is the correct, further investigations will be necessary considering cortical analysis, top down processing and multisensory modeling.

Two competing hypotheses have been proposed to explain the neural mechanisms of multisensory activation after visual deprivation (Amedi et al., 2007; Striem-Amit et al., 2012; Ortiz-Terán et al., 2017; Chebat et al., 2018): the “rewiring hypothesis” suggests that cross-modal brain responses are mediated by the formation of new pathways in the sensory deprived brain and the “unmasking hypothesis” suggests that the loss of a sensory input induces unmasking and/or strengthening of the existing neural pathways. Our results support the unmasking hypothesis suggesting that cortical reorganization is a fast process that supports changes of audio space perception after a short period of visual loss. These results may have a strong impact for rehabilitation purposes by using the audio input to improve spatial representation and to stimulate residual visual regions of patients having central scotoma due to Macular Degeneration.

## Data Availability

The datasets generated for this study are available on request to the corresponding author.

## Ethics Statement

This study was approved by the ethics committees of the local health services: Comitato Etico, ASL3 Genovese, Italy. Subjects (both patients and controls) signed written consents prior to performing the experiment.

## Author Contributions

HA, WS, and MG designed the experiment. HA and WS performed the experiment and analyzed the data with CC. All authors contributed to the writing and revising of the manuscript.

## Conflict of Interest Statement

The authors declare that the research was conducted in the absence of any commercial or financial relationships that could be construed as a potential conflict of interest.

## Abbreviations

CR: central responses
MD: macular degeneration
PR: peripheral responses.

## References

[B1] Abboud S. Cohen L. (2018). Distinctive interaction between cognitive networks and the visual cortex in early blind individuals. *bioRxiv* 437988. 10.1093/cercor/bhz006 30715236

[B2] Alais D. Burr D. (2004). The ventriloquist effect results from near-optimal bimodal integration. *Curr. Biol.* 14 257–262. 10.1016/s0960-9822(04)00043-0 14761661

[B3] Amedi A. Stern W. M. Camprodon J. A. Bermpohl F. Merabet L. Rotman S. (2007). Shape conveyed by visual-to-auditory sensory substitution activates the lateral occipital complex. *Nat. Neurosci.* 10 687–689. 10.1038/nn1912 17515898

[B4] Chebat D.-R. Harrar V. Kupers R. Maidenbaum S. Amedi A. Ptito M. (2018). “Sensory substitution and the neural correlates of navigation in blindness,” in *Mobility of Visually Impaired People*, eds Pissaloux E. Velazquez R. (Cham: Springer), 167–200. 10.1007/978-3-319-54446-5_6

[B5] Chen F. K. Patel P. J. Xing W. Bunce C. Egan C. Tufail A. T. (2009). Test–retest variability of microperimetry using the Nidek MP1 in patients with macular disease. *Invest. Ophthalmol. Vis. Sci.* 50 3464–3472. 19324853 10.1167/iovs.08-2926

[B6] Cohen Y. E. Andersen R. A. (2002). A common reference frame for movement plans in the posterior parietal cortex. *Nat. Rev. Neurosci.* 3 553–562. 10.1038/nrn873 12094211

[B7] Collignon O. Champoux F. Voss P. Lepore F. (2011). Sensory rehabilitation in the plastic brain. *Prog. Brain Res.* 191 211–231. 10.1016/B978-0-444-53752-2.00003-5 21741554

[B8] Collignon O. Charbonneau G. Peters F. Nassim M. Lassonde M. Lepore F. (2013). Reduced multisensory facilitation in persons with autism. *Cortex* 49 1704–1710. 10.1016/j.cortex.2012.06.001 22818902

[B9] Collignon O. Voss P. Lassonde M. Lepore F. (2009). Cross-modal plasticity for the spatial processing of sounds in visually deprived subjects. *Exp. Brain Res.* 192 343–358. 10.1007/s00221-008-1553-z 18762928

[B10] Doksum K. (1974). Empirical probability plots and statistical inference for nonlinear models in the two-sample case. *Ann. Stat.* 2 267–277. 10.1214/aos/1176342662

[B11] Erickson K. I. Colcombe S. J. Wadhwa R. Bherer L. Peterson M. S. Scalf P. E. (2007). Training-induced plasticity in older adults: effects of training on hemispheric asymmetry. *Neurobiol. Aging* 28 272–283. 10.1016/j.neurobiolaging.2005.12.012 16480789

[B12] Fellows I. (2012). {Deducer}: a data analysis GUI for {R}. *J. Stat. Softw.* 49 1–15.

[B13] Finocchietti S. Cappagli G. Gori M. (2015). Encoding audio motion: spatial impairment in early blind individuals. *Front. Psychol.* 6:1357. 10.3389/fpsyg.2015.01357 26441733 PMC4561343

[B14] Gori M. Vercillo T. Sandini G. Burr D. (2014). Tactile feedback improves auditory spatial localization. *Front. Psychol.* 5:1121. 10.3389/fpsyg.2014.01121 25368587 PMC4202795

[B15] Han B. Comaniciu D. Davis L. (2004). “Sequential kernel density approximation through mode propagation: applications to background modeling,” in *Proceedings of the 6th Asian Conference on Computer Vision*, Jeju 813–818.

[B16] Harrar V. Aubin S. Chebat D.-R. Kupers R. Ptito M. (2018). “The multisensory blind brain,” in *Mobility of Visually Impaired People*, eds Pissaloux E. Velazquez R. (Cham: Springer), 111–136. 10.1007/978-3-319-54446-5_4

[B17] Hassan S. E. Lovie-Kitchin J. E. Woods R. L. (2002). Vision and mobility performance of subjects with age-related macular degeneration. *Optom. Vis. Sci.* 79 697–707. 10.1097/00006324-200211000-00007 12462538

[B18] Heiting G. (2017). *Multifocal Contact Lenses.* Available at: https://www.allaboutvision.com/over40/multifocalcls.htm (accessed June 16, 2019).

[B19] Jay M. F. Sparks D. L. (1984). Auditory receptive fields in primate superior colliculus shift with changes in eye position. *Nature* 309 345–347. 10.1038/309345a0 6727988

[B20] Keil J. Senkowski D. (2018). Neural oscillations orchestrate multisensory processing. *Neuroscientist* 24 609–626. 10.1177/1073858418755352 29424265

[B21] King A. J. (2009). Visual influences on auditory spatial learning. *Philos. Trans. R. Soc. Lond. B Biol. Sci.* 364 331–339. 10.1098/rstb.2008.0230 18986967 PMC2674475

[B22] Kramer A. F. Bherer L. Colcombe S. J. Dong W. Greenough W. T. (2004). Environmental influences on cognitive and brain plasticity during aging. *J. Gerontol. A Biol. Sci. Med. Sci.* 59 M940–M957. 15472160 10.1093/gerona/59.9.m940

[B23] Kramer A. F. Erickson K. I. (2007). Capitalizing on cortical plasticity: influence of physical activity on cognition and brain function. *Trends Cogn. Sci.* 11 342–348. 10.1016/j.tics.2007.06.009 17629545

[B24] Lessard N. Paré M. Lepore F. Lassonde M. (1998). Early-blind human subjects localize sound sources better than sighted subjects. *Nature* 395 278–280. 10.1038/26228 9751055

[B25] Lund R. D. (1985). *Development & Plasticity of the Brain.* Oxford: Oxford University Press.

[B26] Martuzzi R. Murray M. M. Michel C. M. Thiran J.-P. Maeder P. P. Clarke S. (2006). Multisensory interactions within human primary cortices revealed by BOLD dynamics. *Cereb. Cortex* 17 1672–1679. 10.1093/cercor/bhl077 16968869

[B27] Mercier M. R. Foxe J. J. Fiebelkorn I. C. Butler J. S. Schwartz T. H. Molholm S. (2013). Auditory-driven phase reset in visual cortex: human electrocorticography reveals mechanisms of early multisensory integration. *Neuroimage* 79 19–29. 10.1016/j.neuroimage.2013.04.060 23624493 PMC3677511

[B28] Ortiz-Terán L. Diez I. Ortiz T. Perez D. L. Aragón J. I. Costumero V. (2017). Brain circuit–gene expression relationships and neuroplasticity of multisensory cortices in blind children. *Proc. Natl. Acad. Sci. U.S.A.* 114 6830–6835. 28607055 10.1073/pnas.1619121114 PMC5495230

[B29] Pouget A. Deneve S. Duhamel J. R. (2002). A computational perspective on the neural basis of multisensory spatial representations. *Nat. Rev. Neurosci.* 3 741–747. 10.1038/nrn914 12209122

[B30] Rauschecker J. P. (1995). Developmental plasticity and memory. *Behav. Brain Res.* 66 7–12. 10.1016/0166-4328(94)00117-x 7755902

[B31] Röder B. Kusmierek A. Spence C. Schicke T. (2007). Developmental vision determines the reference frame for the multisensory control of action. *Proc. Natl. Acad. Sci. U.S.A.* 104 4753–4758. 10.1073/pnas.0607158104 17360596 PMC1838672

[B32] Romei V. Murray M. M. Cappe C. Thut G. (2009). Preperceptual and stimulus-selective enhancement of low-level human visual cortex excitability by sounds. *Curr. Biol.* 19 1799–1805. 10.1016/j.cub.2009.09.027 19836243

[B33] Rousselet G. A. Pernet C. R. Wilcox R. R. (2017). Beyond differences in means: robust graphical methods to compare two groups in neuroscience. *Eur. J. Neurosci.* 46 1738–1748. 10.1111/ejn.13610 28544058

[B34] Santangelo V. Macaluso E. (2012). “Spatial attention and audiovisual processing,” in *The New Handbook of Multisensory Processing*, ed. Stein B. E. (Cambridge, MA: MIT Press), 359–370.

[B35] Schuchard R. A. (2005). Preferred retinal loci and macular scotoma characteristics in patients with age-related macular degeneration. *Can. J. Ophthalmol.* 40 303–312. 10.1016/s0008-4182(05)80073-0 15947800

[B36] Stein B. E. (2012). *The New Handbook of Multisensory Processing.* Cambridge, MA: Mit Press.

[B37] Striem-Amit E. Cohen L. Dehaene S. Amedi A. (2012). Reading with sounds: sensory substitution selectively activates the visual word form area in the blind. *Neuron* 76 640–652. 10.1016/j.neuron.2012.08.026 23141074

[B38] Sunness J. S. Applegate C. A. Haselwood D. Rubin G. S. (1996). Fixation patterns and reading rates in eyes with central scotomas from advanced atrophic age-related macular degeneration and Stargardt disease. *Ophthalmology* 103 1458–1466. 10.1016/s0161-6420(96)30483-1 8841306 PMC2730505

[B39] Vercillo T. Milne J. L. Gori M. Goodale M. A. (2015). Enhanced auditory spatial localization in blind echolocators. *Neuropsychologia* 67 35–40. 10.1016/j.neuropsychologia.2014.12.001 25484307

[B40] Voss P. (2018). Brain (re) organization following visual loss. *Wiley Interdiscip. Rev. Cogn. Sci.* 10:e1468. 10.1002/wcs.1468 29878533

[B41] Voss P. Lassonde M. Gougoux F. Fortin M. Guillemot J.-P. Lepore F. (2004). Early-and late-onset blind individuals show supra-normal auditory abilities in far-space. *Curr. Biol.* 14 1734–1738. 10.1016/j.cub.2004.09.051 15458644

[B42] Voss P. Zatorre R. J. (2012). Organization and reorganization of sensory-deprived cortex. *Curr. Biol.* 22 R168–R173. 10.1016/j.cub.2012.01.030 22401900

[B43] Wheeler B. Torchiano M. (2010). *lmPerm: Permutation Tests for Linear Models. R Package Version, 1(1.2).* Available at: https://CRAN.R-project.org/package=lmPerm

[B44] Wilcox R. R. Erceg-Hurn D. M. (2012). Comparing two dependent groups via quantiles. *J. Appl. Stat.* 39 2655–2664. 10.1080/02664763.2012.724665

